# A Comparative, Multicenter, Prospective, Randomized Study to Evaluate the Efficacy, Safety, and Delay of Relapse of Ceramide-Based Post-biotic Moisturizer Versus Paraffin-Based Moisturizer in Mild to Moderate Atopic Dermatitis

**DOI:** 10.7759/cureus.76762

**Published:** 2025-01-01

**Authors:** Abhishek De, Saswati Halder, Amit Madan, Uday Kiran Raja, Patnala Guru Prasad, Dhiraj Dhoot, Gaurang Jani, Tripti Sharma

**Affiliations:** 1 Dermatology, Wizderm Speciality Skin and Hair Clinic, Kolkata, IND; 2 Dermatology, Calcutta School of Tropical Medicine, Kolkata, IND; 3 Dermatology, Ajanta Research Centre, Lucknow, IND; 4 Dermatology, Narayana Medical College, Nellore, IND; 5 Dermatology, King George Hospital, Visakhapatnam, IND; 6 Global Medical Affairs, Glenmark Pharmaceuticals Ltd., Mumbai, IND; 7 Dermatology, Glenmark Pharmaceuticals Ltd., Mumbai, IND

**Keywords:** atopic dermatitis, ceramide moisturizers, moisturizers, post-biotic moisturizers, relapse in ad, remission in ad

## Abstract

Background: Atopic dermatitis (AD) is a chronic relapsing inflammatory disease that impacts the quality of life of affected individuals as well as their families. Its pathogenesis involves impaired skin barrier function and immune dysregulation. Moisturizers are used in AD management as they help in repairing the skin barrier.

Objective: The objective of this study was to compare the efficacy, safety, and prevention of relapse with ceramide-based post-biotic moisturizer against paraffin-based moisturizer in mild to moderate AD.

Method: A total of 160 AD patients were grouped into two: Group I received desonide cream 0.05% and ceramide-based post-biotic moisturizer and Group II received desonide cream 0.05% and paraffin-based moisturizer. Both groups were given treatment for four weeks. Patients who achieved complete resolution entered the maintenance phase for a maximum duration of three months. They were followed up every two weeks telephonically or as and when the patient experienced a relapse.

Results: All 160 patients were completely cured and entered the maintenance phase at week 4. A total of 96/160 (71.25%) patients relapsed, with 44 relapses in Group I (55%) and 52 (65%) in Group II (p=0.25). However, in terms of mean relapse time, Group I had a 72.52±15.01 day remission period, whereas Group II had a 47.44±21.49 day remission period (p=0.0001). Moreover, Group I showed a statistically significantly prolonged estimated median time to relapse compared with Group II (median: 85 days versus 71 days, p=0.05). Both moisturizers were tolerated very well.

Conclusion: Although both moisturizers were effective in resolving symptoms in the treatment phase, the ceramide-based post-biotic moisturizer was more effective and statistically significant in extending the remission period against the paraffin-based moisturizer in patients with mild to moderate AD.

## Introduction

Atopic dermatitis (AD) is a chronic inflammatory relapsing skin disorder that can significantly impact the quality of life of affected individuals as well as their families. Its pathogenesis is a little complex, but it is associated with impaired skin barrier function and immune dysregulation [1-3]. Currently, AD is very well managed with several therapeutic approaches, which include nurturing skin hydration, avoiding allergens, and using topical anti-inflammatory drugs such as corticosteroids or topical calcineurin inhibitors. However, while all these therapies can alleviate the symptoms, their use is not effective enough, and hence, the recurrence rate is on the higher side [1,4]. Most of the clinical studies on AD have been focused on acute control, lacking long-term clinical control.

Moisturizers restore the barrier disruption and decrease the requirement for topical corticosteroids [4-6]. Recently, the interplay between skin immunity and commensal microbes has led to a number of clinical research investigating the safety and efficacy of probiotics for topical use in AD [7]. Probiotics are live microorganisms conferring health benefits [8], while post-biotics are functional bioactive compounds, which are generated in a matrix during fermentation. Both probiotics and post-biotics may be used to promote health [9]. There are currently many reported and active clinical trials using topical probiotics for AD.

Currently, for the management of AD, many types of moisturizers are used, namely, paraffin-based moisturizers and ceramide-based moisturizers. To add to this list, a ceramide-based post-biotic moisturizer was commercialized in India in 2022. There are studies showing the efficacy of moisturizers in AD [10], but comparative clinical studies are scarce [11]. Hence, this study was conducted to evaluate the efficacy, safety, and prevention of relapse with ceramide-based post-biotic moisturizer against paraffin-based moisturizer in mild to moderate AD.

## Materials and methods

Setting

This was a multicenter, prospective, blinded, randomized comparative study examining the efficacy, safety, and prevention of relapse of a ceramide-based post-biotic moisturizer against paraffin-based moisturizer in children (>3 months) and adults with mild to moderate atopic dermatitis (Investigator Global Assessment (IGA) score < 3) [2]. Patients not deemed fit to be prescribed topical steroids and emollients and with severe systemic illness (chronic infections, autoimmune disorders, or malignancies) or receiving any systemic immunosuppressive therapy were excluded. This study (registered with the Clinical Trials Registry - India (registration number: CTRI/2023/03/050333)) was carried out at five centers in India with prior approval from the ethics committee for respective centers from March 2023 to January 2024 and in accordance with Good Clinical Practices and the 1996 Declaration of Helsinki.

Sample size

With a maximal type I error of 5% and assuming a standard deviation of 10.7, a sample size of 72 in each group (144 in total), at a 1:1 ratio, is needed to achieve a power of 80% to detect a mean difference of 5. Incorporating a dropout rate of 10%, a total of 160 patients (80 in each group) were included.

Study design

One hundred sixty patients fulfilling the selection criteria were randomized to two parallel treatment groups by computer randomization method, and each group had 80 patients (allocation ratio: 1:1). Written informed consent was obtained either in English or Hindi, or regional language, per the patient choice. Diagnoses were made on clinical examination and diagnosed patients' demographics and baseline clinical characteristics, including the duration of current illness recorded at the first visit. Additionally, Dermatology Life Quality Index (DLQI) score was recorded at the same visit and end of treatment [2].

The study was conducted in two phases, with phase 1 being the treatment phase and phase 2 being the maintenance phase. In the treatment phase, the included patients were divided into two groups, where Group I received topical corticosteroid (desonide cream 0.05%) and ceramide-based post-biotic moisturizer to be applied as required and Group II received topical corticosteroid (desonide cream 0.05%) and paraffin-based moisturizer. Both groups received the given treatment for a maximum duration of four weeks. During the four-week treatment phase, the topical corticosteroid (desonide cream 0.05%) was given twice daily for two weeks, followed by once daily for one week, followed by alternate days for one week, and was applied on lesions (dry itchy erythematous areas with or without oozing). The amount of topical corticosteroid applied was to be one fingertip unit for every two palm areas of lesional skin.

Either of the moisturizers was applied twice daily, once after bathing on damp skin and once in the evening. They were given a 400 g moisturizer (four jars of 100 g) per month since the recommended amount was 100 g/week, and compliance was ensured by asking them to return empty jars at every follow-up visit. The moisturizer was applied first, and desonide was applied after a 30-minute gap. During the entire study period, patients were not allowed to take any other medication for AD.

**Figure 1:**
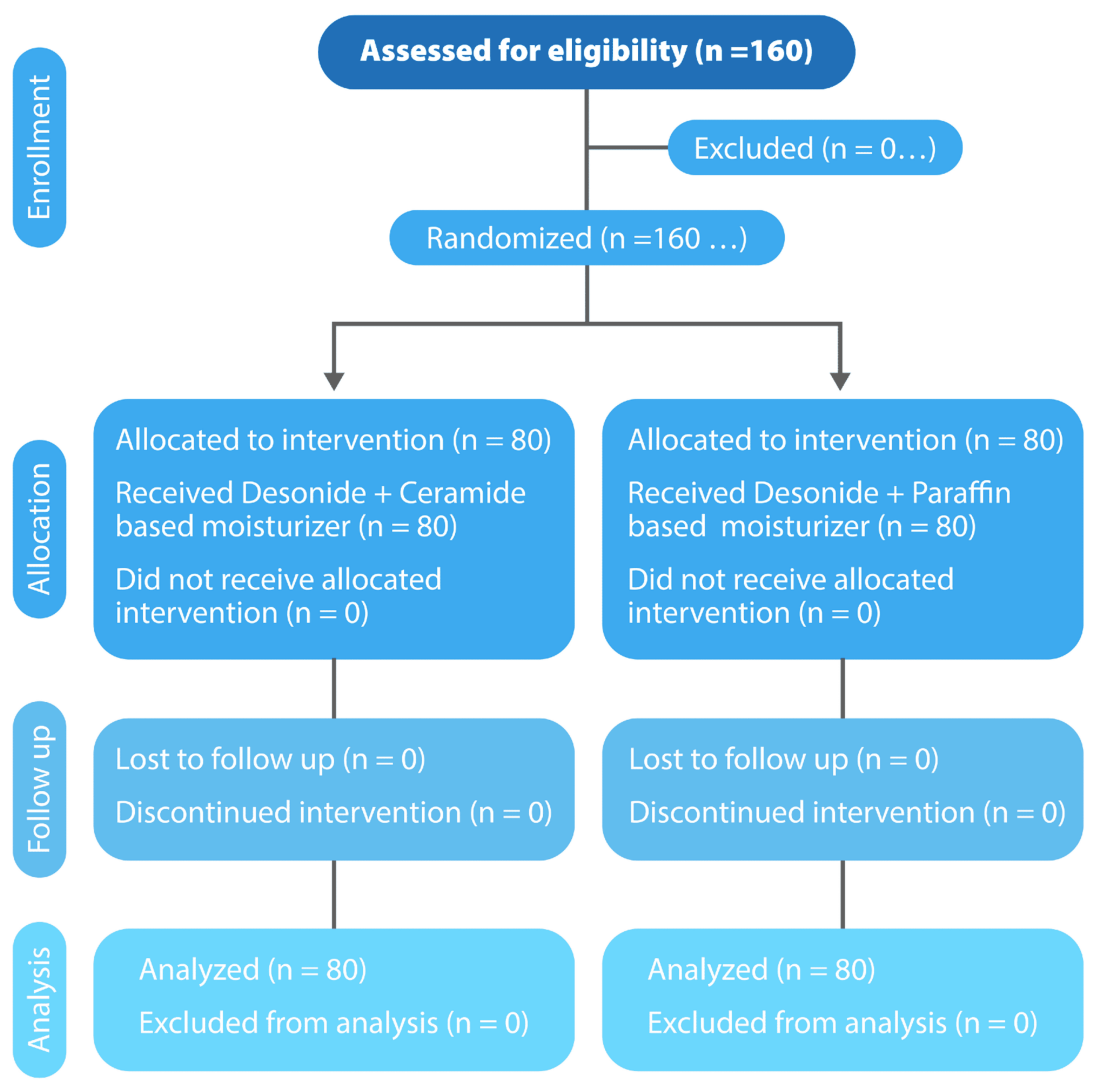
CONSORT diagram CONSORT: Consolidated Standards of Reporting Trials

The patients who achieved complete resolution then entered the maintenance phase for a maximum duration of three months. They were followed up every two weeks telephonically or as and when the patient experienced a relapse. During the maintenance phase, both groups continued on their respective moisturizers, i.e., Group I on ceramide-based post-biotic moisturizer and Group II on paraffin-based moisturizer twice daily. Those who failed to achieve resolution at the end of the treatment phase were continued on treatment according to the physician's discretion and were left out of the analysis. Both moisturizers were supplied by Glenmark Pharmaceuticals Ltd., India. The complete study design is shown in Figure 1.

Measurement of treatment effect

The primary end point of the study was to compare the percentage of patients achieving a complete resolution at four weeks (IGA score of clear or almost clear and ≥2 point reduction from baseline) and the percentage of patients who suffered from relapse (relapse was defined as recurrence of either of AD symptoms such as erythema, edema, excoriation, and lichenification, except itching) in both groups during the course of the study. Additionally, time to resolution and relapse and improvement in AD (assessed by different scores) at different time intervals were also assessed.

Efficacy based on the Eczema Area and Severity Index (EASI) assessment [2], Visual Analog Scale (VAS) itching score [12], and Investigator Global Assessment (IGA) score [2] was assessed at two and four weeks, and quality of life was assessed using the Dermatology Life Quality Index (DLQI) [2] at four weeks.

Analysis set

The full analysis set (FAS) (i.e., those patients who received at least one dose of medication and completed at least one post-baseline follow-up visit) was considered for effectiveness and safety analyses.

Statistical analysis

Baseline variables were presented as numbers and percentages, and as mean with standard deviations (SDs) depending on the distribution of data. The difference in the proportion of patients with severity scores (based on improvement criteria) was analyzed using the Mann-Whitney U test with a significance level set at 0.05, as appropriate. The time to relapse end point was calculated as the time from entry into the maintenance phase until a relapse occurred. Kaplan-Meier estimates were calculated to describe the time to relapse distribution, and the relapse rate was compared using the Chi-square test. All p-values were two-tailed. The denominator for the calculation of the relapse rate was the number of patients who had achieved a cure. All statistical analyses were performed using SPSS version 16.0 (SPSS Inc., Chicago, Ill) and Microsoft Excel (Microsoft Corp., Redmond, WA).

## Results

Patients

Of the 160 patients enrolled in this study, all were included in the FAS. Out of 160 patients, 74 (46.25%) patients were <18 years of age, while 86 (53.75%) were >18 years. All baseline characteristics are presented in Table 1. The patient distribution was homogeneous in all groups, and most patients presented with mild to moderate AD.

**Table 1:** Demographic information of all patients in both groups IGA: Investigator Global Assessment, EASI: Eczema Area and Severity Index, VAS: Visual Analog Scale, DLQI: Dermatology Life Quality Index, NA: not applicable, SD: standard deviation

Characteristics	Ceramide post-biotic group	Paraffin group	p-value
Age (years), mean±SD	27.16±18.49	25.81±17.47	0.63
Male (n=85)	45 (56.25)	40 (50)	NA
Female (n=75)	35 (43.75)	40 (50)	
Duration of disease (months), mean±SD	0.36±1.48	0.38±1.50	0.93
IGA, mean±SD	2.50±0.50	2.51±0.50	1
EASI, mean±SD	7.23±3.82	7.44±4.06	0.73
VAS for itching, mean±SD	3.80±1.52	3.79±1.36	0.96
DLQI, mean±SD	5.26±2.27	5.13±2.59	0.73

Treatment response

Complete Resolution of Symptoms and Improvement in Mean Scores

At week 4, all 160 patients were completely cured and entered the maintenance phase. Moreover, at week 2 only, 29/80 (36.25%) patients in Group I and 28/80 (35%) patients in Group II achieved complete resolution, and the rest achieved the same at week 4 (Table 2). There was no significant statistical difference between the groups at these time intervals.

**Table 2:** Effectiveness scores in terms of mean IGA, EASI, VAS, and DLQI at week 2 and 4 IGA: Investigator Global Assessment, EASI: Eczema Area and Severity Index, VAS: Visual Analog Scale, DLQI: Dermatology Life Quality Index, NA: not applicable, SD: standard deviation

Characteristics	Groups	14 days	28 days
Mean IGA score, mean±SD	Group I	1.19±0.93	0.69±0.47
	Group II	1.33±0.92	0.65±0.48
	p-value	0.34	0.59
Mean EASI score, mean±SD	Group I	2.51±2.42	0.52±0.38
	Group II	2.63±2.60	0.53±0.40
	p-value	0.76	0.87
Mean VAS, mean±SD	Group I	1.65±1.46	1.42±0.83
	Group II	1.92±1.32	1.39±0.80
	p-value	0.22	0.81
Mean DLQI score, mean±SD	Group I	NA	1.48±1.12
	Group II		1.44±1.40
	p-value		0.84
Complete resolution of symptoms, number (%)	Group I	29	51
	Group II	28	52
	p-value	1	1

In terms of IGA score, the mean IGA score reduced to 0.69±0.47 from 2.50±0.50 in Group I, while in Group II, it reduced to 0.65±0.48 from 2.51±0.50 (p=0.59). Similarly, the mean EASI score also reduced to 0.52±0.38 from 7.23±3.82 in Group I and 0.53±0.40 from 7.44±4.06 in Group II (p=0.87). Similar results were reported for mean VAS and DLQI scores as shown in Table 2.

Relapse Rates

**Figure 2:**
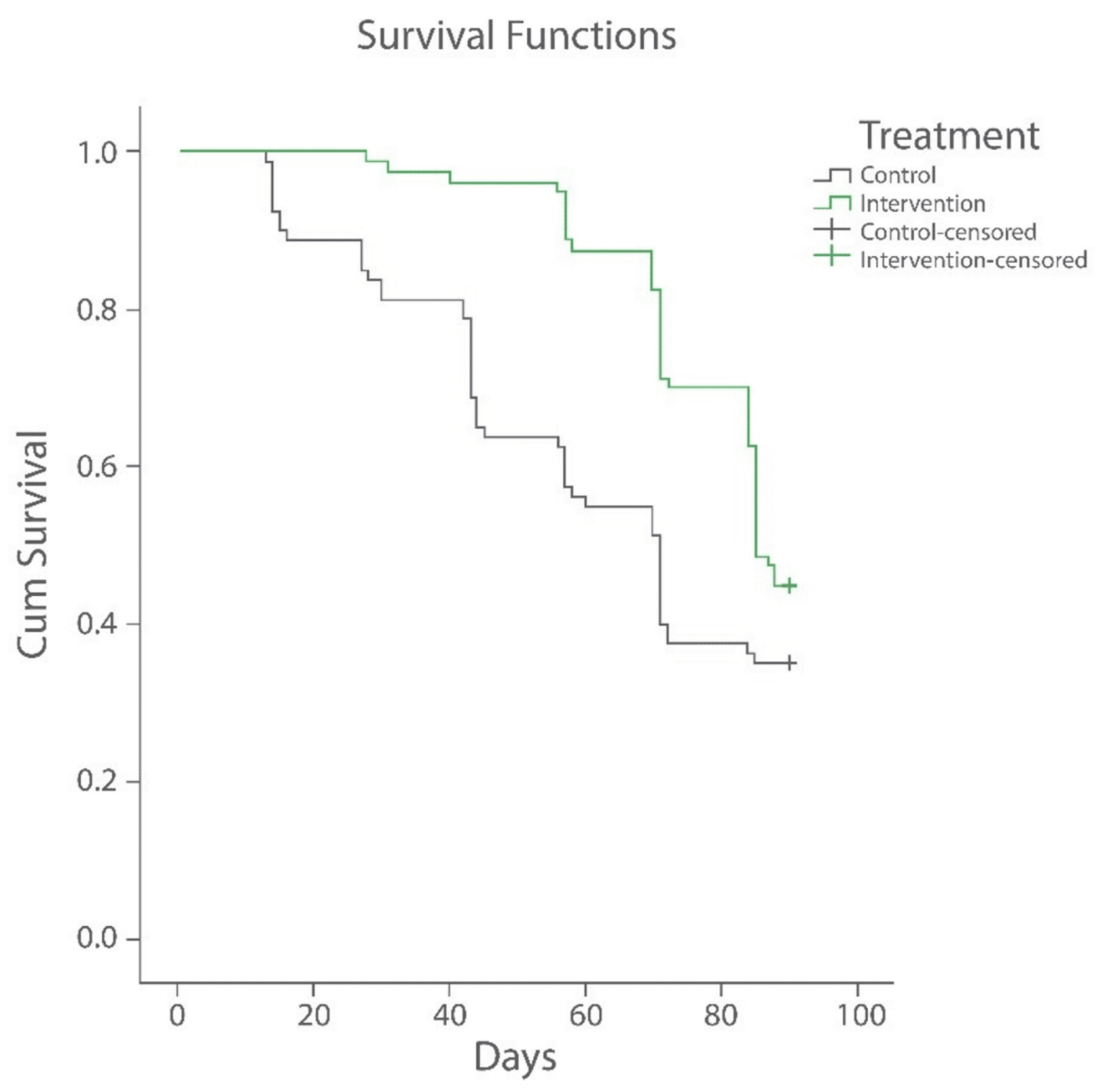
Kaplan-Meier plot for time to relapse The function shows the proportion of patients with relapse at the given time point.

A total of 96/160 (71.25%) patients relapsed following successful study completion. There were 44 (55%) relapses in Group I and 52 (65%) in Group II; the difference in relapse rates among the groups was not statistically significant (p=0.25). However, in terms of mean relapse time, Group I had a 72.52±15.01 day remission period, whereas Group II had a 47.44±21.49 day remission period, which was statistically significant (p=0.0001) (Table 3). Additionally, ceramide-based post-biotic moisturizer showed a statistically significantly prolonged estimated median time to relapse compared with the paraffin-based moisturizer (median: 85 days versus 71 days, p=0.05) (Figure 2).

**Table 3:** Effectiveness in terms of relapse in both groups at the end of the study SD: standard deviation

Characteristics	Group I	Group II	p-value
Patients with no relapse, number (%)	36 (45)	28 (35)	0.25
Mean time of relapse in days, mean±SD	72.52±15.01	47.44±21.49	0.0001

Safety outcome

Two patients in Group I developed dryness during the maintenance phase after eight weeks, which was mild in nature and did not require any intervention and resolved over the course of therapy. There were no other adverse events reported related to any of the moisturizers.

## Discussion

The use of moisturizers reduces the need for corticosteroids, enhances the clearance of eczema, and prolongs clinical improvement after therapy discontinuation.

In the management of AD, moisturizers not only reduce the need for corticosteroids [6] but also prolong the clinical improvement after discontinuation of therapy by improving skin barrier function. Moisturizers enhance the clearance of eczema [13,14] by reducing trans-epidermal water loss (TEWL) and maintaining hydration in the skin, which is commonly increased in AD. Paraffin-based moisturizers are commonly prescribed in AD, which are occlusive in nature and work by creating a physical barrier on the skin to prevent water loss. Additionally, ceramide-based moisturizers gained attention as they replace natural ceramides in the skin, which are lost in AD [15,16]. Recently, numerous research has been conducted on topical probiotics and post-biotics in dermatology [7,9,17,18]. In this regard, ceramide-based post-biotic moisturizers were commercialized in India.

Multiple studies have proven the efficacy of paraffin-based moisturizers and ceramide-based moisturizers in the management of AD. However, there is a paucity of clinical studies for ceramide-based post-biotic moisturizers, and there is no comparative study between this moisturizer and paraffin-based moisturizer. Hence, this study was conducted to study the prolongation of the remission period between paraffin-based and ceramide-based post-biotic moisturizers in AD.

In this study, our primary objective was to compare the proportion of patients showing complete resolution of AD symptoms in the treatment phase as well as the percentage of patients presenting with relapse in the maintenance phase in both groups. During the treatment phase, all patients achieved complete resolution in both groups, indicating the effectiveness of both moisturizers. This may be due to the concomitant use of topical corticosteroids during this phase. However, during the maintenance phase, where all patients maintained only both moisturizers, a statistical difference was noted in the remission period, indicating a better effect with ceramide-based post-biotic moisturizer. In this group, the estimated median time to relapse was recorded as 85 days compared to 71 days with the paraffin-based moisturizer (p=0.05).

In the study by Gupta et al., no difference was found between ceramide-based and paraffin-based moisturizers in prolonging the remission period [11]. Additionally, in the study by Tiplica et al., after eight weeks, 63.1% of the patients had complete remission with glycerol-based emollient, 41.3% in the no-emollient group, and 51.3% with paraffin-based emollient [19].

The delay in eczema relapse between the two moisturizers in the present study suggests the barrier-strengthening property of topical probiotics/post-biotics.

Multiple research articles have demonstrated the immunomodulatory effect of probiotics in dermatology [4,11,20]. Probiotics are live microorganisms that have a positive influence on the host organism by competing with pathogenic bacteria [17]. However, due to their sensitivity to humidity, temperature, and air conditioning, multiple factors can affect their quality during storage or delivery [21]. Some alternatives such as prebiotics and post-biotics have been suggested. Post-biotic is a non-viable probiotic with immunomodulation ability.

As per the International Scientific Association of Probiotics and Prebiotics, the term "post-biotic" means "a preparation of inanimate microorganisms and/or their components that confers a health benefit on the host" [22]. Post-biotics have several advantages over probiotics, such as defined chemical composition with no antibiotic resistance, permitting their use in immunosuppressed people. Additionally, post-biotic is more stable over a wide range of temperatures with longer shelf life [23]. Moreover, post-biotic is an innovative cosmetic ingredient, since there is no need to maintain viable cells in formulations [18].

Hoang et al. [24] and Inoue et al. [25] demonstrated significant improvement in AD symptoms in adults and children with heat-inactivated *Lactobacillus acidophilus* L-92 strain. In these studies, the improvement is associated with the suppression of Th2-dominant inflammation. Moreover, several other studies have also demonstrated the potential benefits of post-biotics [26-29]. Our study also demonstrated a significant delay in relapse with the ceramide-based post-biotic moisturizer, and it was found to be a better moisturizer than the paraffin-based moisturizer.

Limitation of the study

This study has a small sample size; hence, a large-scale clinical study is warranted to further substantiate these results.

## Conclusions

Our study suggested that both moisturizers were effective in resolving symptoms in the treatment phase. However, the ceramide-based post-biotic moisturizer was more effective and statistically significant in extending the remission period in contrast to paraffin-based moisturizer in patients with mild to moderate AD. In particular, the potential decrease in the likelihood of a relapse could have significant implications for physicians when deciding on treatment plans for patients requiring extended periods of care.
